# Preventive Effect of Garlic Oil and Its Organosulfur Component Diallyl-Disulfide on Cigarette Smoke-Induced Airway Inflammation in Mice

**DOI:** 10.3390/nu10111659

**Published:** 2018-11-04

**Authors:** Je-Won Ko, Seong-Hun Jeong, Hyung-Jun Kwon, Na-Rae Shin, Yun-Soo Seo, Jong-Choon Kim, In-Sik Shin, Joong-Sun Kim

**Affiliations:** 1 College of Veterinary Medicine (BK21 Plus Project Team), Chonnam National University, 27 Yongbong-ro, Buk-gu, Gwangju 61186, Korea; rheoda@gmail.com (J.-W.K.); tlsskfo870220@gmail.com (N.-R.S.); toxkim@jnu.ac.kr (J.-C.K.); 2 Namhae Garlic Research Institute, 2465-8 Namhae-daero, Namhae-gun, Gyungnam 52430, Korea; poi1977@hanmail.net; 3 Natural Product Research Center, Korea Research Institute of Bioscience and Biotechnology, 181 Ipsin-gil, Jeongeup-si, Jeonbuk 56212, Korea; hjkwon@kribb.re.kr; 4 Herbal Medicine Resources Research Center, Korea Institute of Oriental Medicine, 177 Geonjae-ro, Naju-si, Jeollanam-do 58245, Korea; sys0109@kiom.re.kr

**Keywords:** garlic oil, diallyl disulfide, cigarette smoke, airway inflammation, extracellular signal-regulated kinase, matrix metalloproteinase-9

## Abstract

Garlic (*Allium sativum*) has traditionally been used as a medicinal food and exhibits various beneficial activities, such as antitumor, antimicrobial, hypolipidemic, antiarthritic, and hypoglycemic activities. The aim of this study was to explore the preventive effect of garlic oil (GO) and its organosulfur component diallyl disulfide (DADS) on cigarette smoke (CS)-induced airway inflammation. Mice were exposed to CS daily for 1 h (equivalent to eight cigarettes per day) for two weeks, and intranasally instilled with lipopolysaccharide (LPS) on day 12 after the initiation of CS exposure. GO and DADS were administered to mice by oral gavage, both at rates of 20 and 40 mg/kg, for 1 h before CS exposure for two weeks. In the bronchoalveolar lavage fluid, GO and DADS inhibited the elevation in the counts of inflammatory cells, particularly neutrophils, which were induced in the CS and LPS (CS + LPS) group. This was accompanied by the lowered production (relative to the CS + LPS group) of interleukin (IL)-1β, IL-6, and tumor necrosis factor-α. Histologically, GO and DADS inhibited the CS- and LPS-induced infiltration of inflammatory cells into lung tissues. Additionally, GO and DADS inhibited the phosphorylation of extracellular signal-regulated kinase and the expression of matrix metalloproteinase-9 in the lung tissues. Taken together, these findings indicate that GO and DADS could be a potential preventive agent in CS-induced airway inflammation.

## 1. Introduction

Cigarette smoke (CS) is considered a crucial risk factor for human health. It is closely associated with the development of various diseases and is particularly regarded as the most important cause of chronic obstructive pulmonary disease (COPD) [1]. CS is composed of numerous chemicals that can cause various pathological conditions, such as oxidative stress and inflammation [2]. Exposure to CS increases the recruitment of inflammatory cells into lung tissues [3]. The recruited inflammatory cells are activated by CS exposure and produce large quantities of proinflammatory mediators, such as reactive oxygen species (ROS), cytokines, and chemokines, which results in the exacerbation of airway inflammation [4,5]. Eventually, chronic CS exposure leads to the loss of normal lung function [6,7]. Currently, many researchers are investigating therapeutics for the treatment of COPD through in vivo and in vitro experiments [8,9].

Garlic (*Allium sativum*) has been traditionally used as a medicinal food for treating many inflammatory diseases and is mainly administered in powder and oil forms. In addition, garlic is commonly used for treating coughs, parasitic infestations, insect bites, and constipation [10]. Recently, it has been revealed that garlic oil (GO) has various pharmacological properties [11,12,13,14] that are closely related to its various active compounds. Of these compounds, organosulfur compounds including diallyl sulfide, diallyl disulfide (DADS), and diallyl trisulfide exhibit many biological activities, such as antioxidant, antitumor, anti-inflammatory, renoprotective, hepatoprotective, and antiobesity activities [15,16,17,18]. Recent studies have reported that organosulfur compounds isolated from garlic can effectively suppress allergic responses in an experimental asthma model [19,20]. However, there are no studies on the preventive effect of GO and DADS against CS-induced pulmonary inflammation.

Therefore, the aim of this study was to explore the preventive effect of GO and DADS on airway inflammation caused by the exposure of mice to CS and lipopolysaccharide (LPS). Furthermore, we investigated the mechanism of the anti-inflammatory actions of GO and DADS by measuring the expression levels of inflammation-related proteins.

## 2. Materials and Methods 

### 2.1. Animals

We used male C57BL/6N mice (6 weeks old, 20–25 g, Koatech Co., Pyeongtaek, Republic of Korea) after 1 week of quarantine and acclimatization. They were housed in polycarbonate cages in a room maintained at 23 ± 3 °C and 50% ± 10% relative humidity. They were provided with artificial lighting from 08:00 to 20:00 and with 13–18 air changes per hour. Mice were provided with tap water that had been sterilized by ultraviolet irradiation and with commercial rodent chow (Samyang Feed Co., Wonju, Korea) ad libitum. All procedures were approved by the Institutional Animal Care and Use Committee of Chonnam National University (Gwangju, Republic of Korea), and were performed in compliance with the Guide for the Care and Use of Laboratory Animals of the National Institute of Health and with Korean national laws on animal welfare.

### 2.2. CS-Induced Airway Inflammation

The mouse model for lung inflammation induced by CS and LPS was described in a previous report [2]. Briefly, CS was generated from 3R4F research cigarettes (Kentucky reference cigarette, University of Kentucky, Lexington, KY, USA) containing 11.0 mg of total particulate matter, 9.4 mg of tar, and 0.76 mg of nicotine per cigarette. The exposure of mice to the CS (1 puff/min, 35 mL puff volume over 2 s, every 60 s, 8 cigarettes per day) was conducted using a cigarette smoke generator (Daehan Biolink, Inchun, Korea). The mice received 1 h of CS exposure in an exposure chamber (50 cm × 30 cm × 30 cm) for 14 days. LPS (10 µg per mouse) was instilled intranasally into anesthetized mice on day 12 after the initiation of CS exposure. GO (20 and 40 mg/kg, Sigma-Aldrich, Carlsbad, CA, USA), DADS (20 and 40 mg/kg, Tokyo Chemical Industry Co., Tokyo, Japan), and roflumilast (ROF) (10 mg/kg, Sigma-Aldrich, Carlsbad, CA, USA) were administered to the mice by daily oral gavage 1 h before CS exposure for 14 days. The test chemicals were dissolved in corn oil, and daily application volumes (5 mL/kg) were calculated based on the most recently recorded body weight of the individual animal. ROF is a phosphodiesterase-4 inhibitor and is recommended for the remedy of COPD [2].

### 2.3. Analysis of Bronchoalveolar Lavage Fluid (BALF)

BALF was collected as described previously [2]. To count inflammatory cells, BALF was stained with Diff-Quik^®^ staining reagent (IMEB Inc., Deerfield, IL, USA). Inflammatory cell counts in the BALF of all the animals were determined by averaging the cell counts from five squares of a slide containing stained samples at a magnification of 400× under a microscope. The levels of interleukin (IL)-6, IL-1β, and tumor necrosis factor (TNF)-α were determined by an enzyme-linked immunosorbent assay (ELISA) (R&D System, Minneapolis, MN, USA). The absorbance was measured at 450 nm.

### 2.4. Histopathology and Immunohistochemistry

Lung tissues were fixed, embedded, sectioned, and stained with hematoxylin and eosin in order to evaluate lung inflammatory response. Each slide was manually evaluated in a completely blinded manner using a light microscope (Leica, Wetzlar, Germany) with 10× and 20× objective lenses and a 100× oil immersion lens. Immunohistochemistry was conducted as previously described [2]. Briefly, each slide was incubated for 10 min at room temperature with 10% goat serum to block nonspecific staining. The slides were incubated overnight with primary mouse matrix metalloproteinase (MMP)-9 antibodies (1:200 dilution, Abcam, Cambridge, MA, USA). After incubating with the primary antibodies, the slides were washed and incubated with biotinylated secondary antibodies at 37 °C for 1 h and then incubated with an avidin-biotin-peroxidase complex (Vector Laboratories, Burlingame, CA, USA) for 1 h at room temperature. The excess complex was removed, and slides were washed with PBS prior to incubating in 0.05% diaminobenzidine (1:200, Millipore Co., Bedford, MA) for 10 min. The slides were counterstained, rinsed with PBS to terminate the reaction, and protected with coverslips prior to microscopic examination.

### 2.5. Quantitative Analysis for Histopathology

Images of 10 randomly selected, non-overlapping areas on each slide were captured by IMTcamCCD5 (IMT i-Solution Inc., Daejeon, Korea). Quantitative analysis of airway inflammation and the evaluation of MMP-9 expression were performed by the IMT i-Solution software (IMT i-Solution Inc., Vancouver, BC, Canada).

### 2.6. Immunoblotting

The preparation of the necessary reagents and the performance of immunoblotting were in accordance with a previous study [21]. The primary antibodies used were as follows: anti-β-actin (Cell Signaling, Danvers, MA, USA), anti-MMP-9 (Abcam), anti-phosphor-extracellular signal-regulated kinase (p-ERK) (Cell Signaling) and anti-ERK (Cell Signaling). The blots were developed using an enhanced chemiluminescence kit (Thermo Scientific, San Diego, CA, USA). Densitometric values of the bands were determined by a ChemiDoc imaging system (Bio-Rad Laboratories, Hercules, CA, USA).

### 2.7. Statistical Analysis

Results are shown as means ± standard deviation (SD). Significance was determined by one-way analysis of variance followed by Dunnett’s adjustment. A *p*-value < 0.05 was considered to be statistically significant.

## 3. Results

### 3.1. Effects of GO and DADS on Body Weight Gains and Inflammatory Cell Counts in BALF

During the study period, similar body weight gains were observed in all groups (normal control (NC): 2.1 ± 0.18 g; CS and LPS (CS + LPS): 1.9 ± 0.21 g; ROF: 2.0 ± 0.19 g; GO-20: 1.9 ± 0.23 g; GO-40: 1.9 ± 0.23 g; DADS-40: 2.0 ± 0.17 g; DADS-20: 2.0 ± 0.14 g).

**Figure 1:**
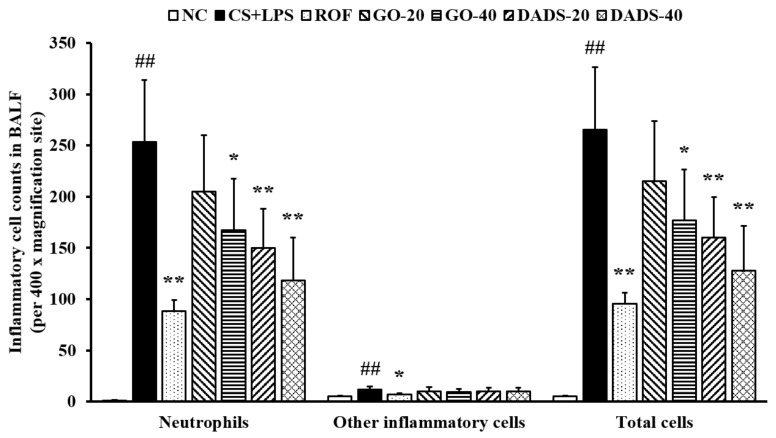
Garlic oil (GO) and diallyl disulfide (DADS) inhibited the number of inflammatory cells in the bronchoalveolar lavage fluid (BALF) of the mice that were exposed to cigarette smoke (CS) and lipopolysaccharide (LPS). Inflammatory cell counts were determined by counting the cells in the BALF of all the animals (*n* = 6 per group) in five squares at 400x magnification under a microscope. NC: normal control; CS + LPS: mice exposed to CS and LPS; ROF: roflumilast (10 mg/kg) administered to mice exposed to CS and LPS; GO-20 and GO-40: GO (20 and 40 mg/kg, respectively) administered to mice exposed to CS and LPS; DADS-20 and DADS-40: DADS (20 and 40 mg/kg, respectively) administered to mice exposed to CS and LPS. Values are expressed as means ± standard deviation (SD) (*n* = 6). Significantly different from NC: ^##^ (*p* < 0.01); significantly different from CS + LPS: *, ** (*p* < 0.05 and < 0.01, respectively).

There was a marked elevation in the counts of inflammatory cells, particularly neutrophils, in the CS + LPS group compared to the normal controls (Figure 1). In contrast, there were substantially lower inflammatory cell counts in the mice treated with GO (40 mg/kg) than in the group of CS + LPS mice. The mice treated with DADS (20 and 40 mg/kg) also exhibited remarkably lower inflammatory cell counts in the BALF than mice of the CS + LPS group.

### 3.2. Effects of GO and DADS on the Production of Proinflammatory Cytokines

**Figure 2:**
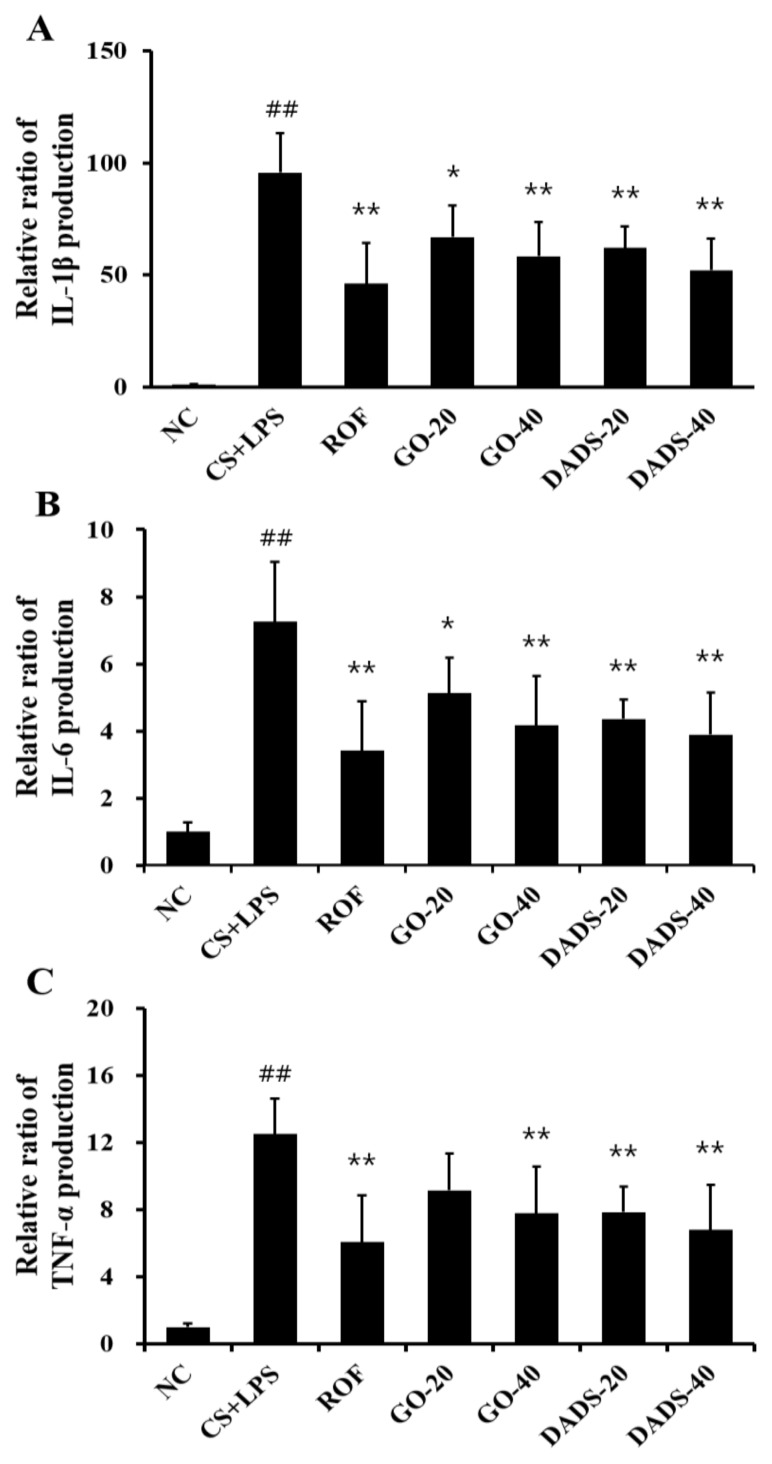
GO and DADS inhibited levels of interleukin (IL)-1β, IL-6, and tumor necrosis factor (TNF)-α in the BALF of mice that were exposed to CS and LPS. (**A**) Level of IL-1 β; (**B**) Level of IL-6; (**C**) Level of TNF-α. Levels of IL-6, IL-1β, and TNF-α were determined using a commercial enzyme-linked immunosorbent assay (ELISA) kit. NC: normal control; CS + LPS: mice exposed to CS and LPS; ROF: ROF (10 mg/kg) administered to mice exposed to CS and LPS; GO-20 and GO-40: GO (20 and 40 mg/kg, respectively) administered to mice exposed to CS and LPS; DADS-20 and DADS-40: DADS (20 and 40 mg/kg, respectively) administered to mice exposed to CS and LPS. Values are expressed as means ± SD (*n* = 6). Significantly different from NC: ^##^ (*p* < 0.01); significantly different from CS + LPS: *, ** (*p* < 0.05 and < 0.01, respectively).

BALF IL-1β concentration was higher in the CS + LPS group than in normal controls (Figure 2A). However, BALF IL-1β concentrations in both GO- and DADS-treated mice were lower than in the CS + LPS group. Similar to the observations of IL-1β concentration, IL-6 and TNF-α concentrations in BALF were markedly higher in the CS + LPS group compared to those in the normal controls. These concentrations were substantially inhibited in mice treated with GO (40 mg/kg) and DADS (20 and 40 mg/kg) compared to the CS + LPS group (Figure 2B,C).

### 3.3. Effects of GO and DADS on the Infiltration of Inflammatory Cells into Lung Tissues

**Figure 3:**
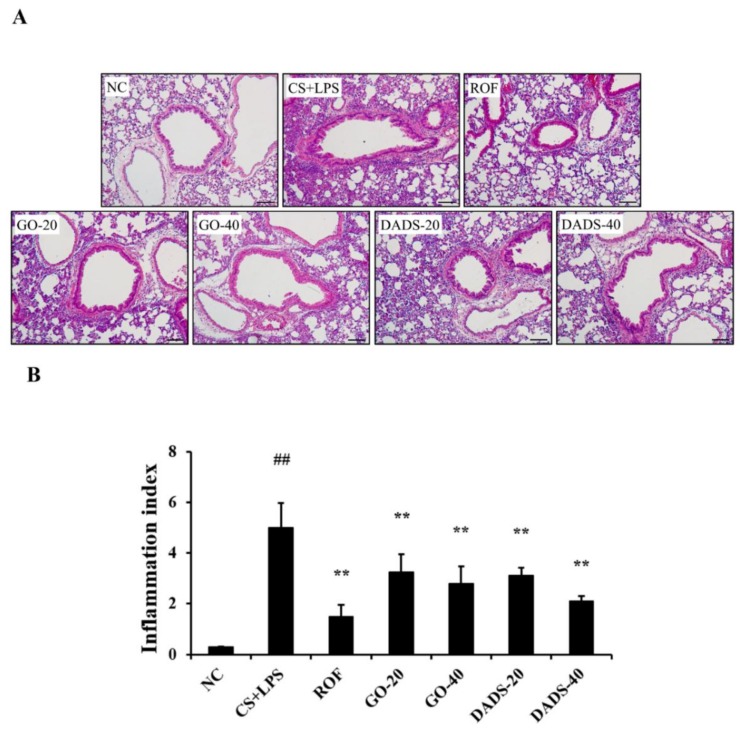
GO and DADS inhibited the accumulation of inflammatory cells in the lung tissues. A representative figure of a peribronchial lesion in lung tissue stained with hematoxylin and eosin (H&E) solution (100× magnification). Scale bar = 50 µm. Quantitative analysis of inflammatory response was performed using IMT i-Solution software (IMT i-Solution Inc.). (**A**) Representative figure of lung tissue; (**B**) quantitative analysis of inflammation. NC: normal control; CS + LPS: mice exposed to CS and LPS; ROF: ROF (10 mg/kg) administered to mice exposed to CS and LPS; GO-20 and GO-40: GO (20 and 40 mg/kg, respectively) administered to mice exposed to CS and LPS; DADS-20 and DADS-40: DADS (20 and 40 mg/kg, respectively) administered to mice exposed to CS and LPS. Values are expressed as means ± SD (*n* = 6). Significantly different from NC: ^##^ (*p* < 0.01); significantly different from CS + LPS: ** (*p* <0.01).

The accumulation of inflammatory cells in the lung tissues increased remarkably in the CS + LPS group in comparison to the normal controls (Figure 3A,B). However, there was a notable inhibition in the accumulation of inflammatory cells in the lung tissues of the mice treated with GO (20 and 40 mg/kg) and DADS (20 and 40 mg/kg) compared to the CS + LPS group.

### 3.4. Effects of GO and DADS on ERK Phosphorylation and MMP-9 Expression

**Figure 4:**
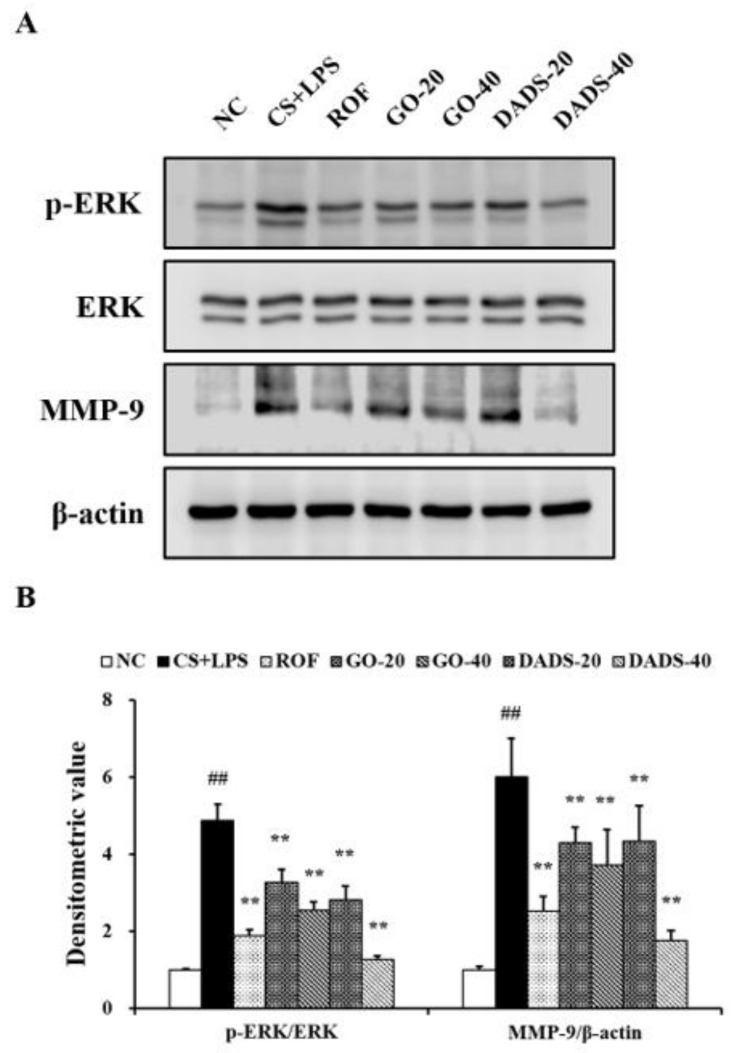
GO and DADS inhibited extracellular signal-regulated kinase (ERK) phosphorylation and matrix metalloproteinase (MMP)-9 expression, which were induced in the CS + LPS group. Protein expression was analyzed by western blot and the relative ratios of protein expression were evaluated. Densitometric values of protein expression were determined using the Chemi-Doc imaging system (Bio-Rad Laboratories). (**A**) Gel images showing protein expression; (**B**) densitometric values of protein expression. NC: normal control; CS + LPS: mice exposed to CS and LPS; ROF: ROF (10 mg/kg) administered to mice exposed to CS and LPS; GO-20 and GO-40: GO (20 and 40 mg/kg, respectively) administered to mice exposed to CS and LPS; DADS-20 and DADS-40: DADS (20 and 40 mg/kg, respectively) administered to mice exposed to CS and LPS. Values are expressed as means ± SD (*n* = 6). Significantly different from NC: ^##^ (*p* < 0.01); significantly different from CS + LPS: ** (*p* < 0.01).

There was a marked elevation in ERK phosphorylation in the lung tissues of the CS + LPS group in comparison to the normal controls (Figure 4A,B). In contrast, ERK phosphorylation was considerably inhibited in the mice treated with GO (20 and 40 mg/kg) and DADS (20 and 40 mg/kg) compared to the CS + LPS group. Consistent with the observations of ERK phosphorylation, MMP-9 expression increased markedly in the CS + LPS group in comparison to the normal controls, whereas it was considerably inhibited in the mice that were treated with GO and DADS compared to the CS + LPS group.

### 3.5. Effects of GO and DADS on MMP-9 Expression in the Lung Tissue

**Figure 5:**
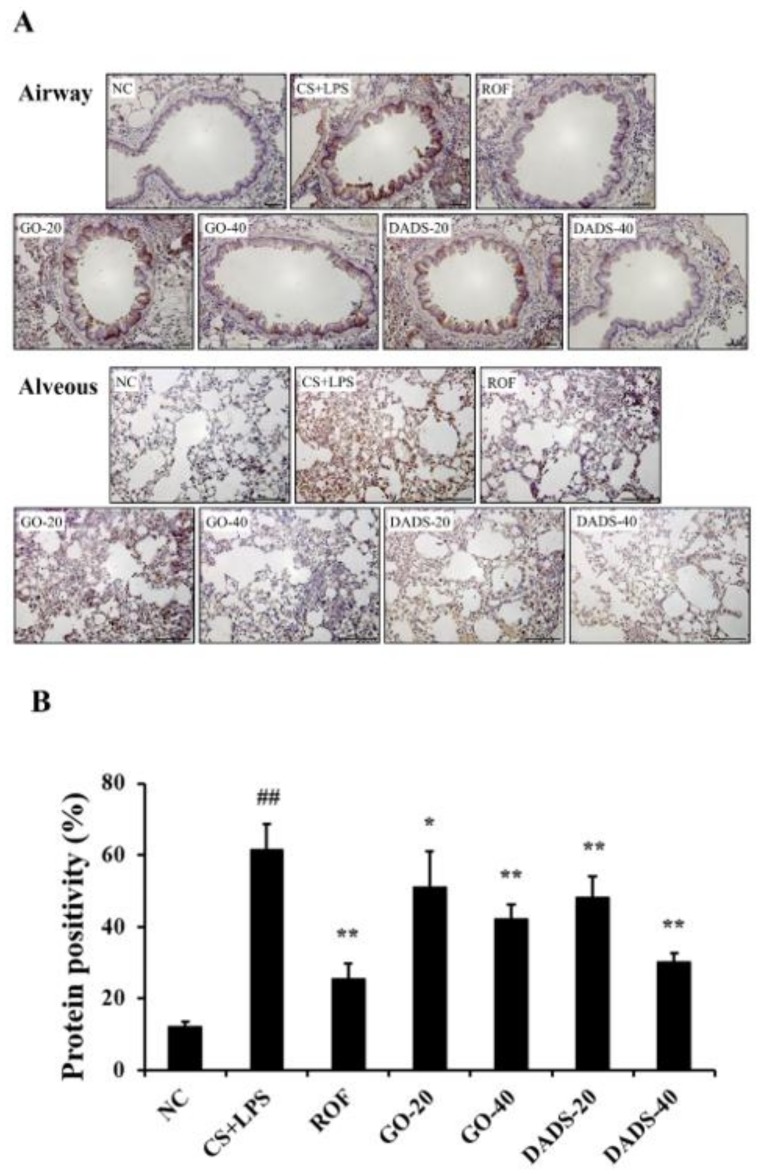
GO and DADS inhibited MMP-9 expression in the lung tissues of the mice, which was induced in the CS + LPS group. Protein expression was analyzed by immunohistochemistry, and values of protein expression were determined using IMT i-Solution software (IMT i-Solution Inc.). Scale bar = 50 µm. (**A**) Representative figure showing protein expression in lung tissue; (**B**) value of MMP-9 expression. NC: normal control; CS + LPS: mice exposed to CS and LPS; ROF: ROF (10 mg/kg) administered to mice exposed to CS and LPS; GO-20 and GO-40: GO (20 and 40 mg/kg, respectively) administered to mice exposed to CS and LPS; DADS-20 and DADS-40: DADS (20 and 40 mg/kg, respectively) administered to mice exposed to CS and LPS. Values are expressed as means ± SD (*n* = 6). Significantly different from NC: ^##^ (*p* < 0.01); significantly different from CS + LPS: *, ** (*p* < 0.05 and < 0.01, respectively).

The CS + LPS group exhibited extensive MMP-9 expression in the lung tissues compared with the normal controls (Figure 5A,B). However, MMP-9 expression had decreased notably in the lung tissues of the GO (20 and 40 mg/kg)-treated mice in comparison to the CS + LPS group. Additionally, there was a substantial reduction in MMP-9 expression in the lung tissues of the DADS (20 and 40 mg/kg)-treated mice in comparison to the CS + LPS group.

## 4. Discussion

Garlic has been used as a medicinal food for a long time because of its various pharmacological properties [22]. Recently, research efforts focusing on the beneficial effects of garlic and its components have increased its importance as a therapeutic agent [23,24]. Our investigation into the preventive effects of GO and DADS on airway inflammation, which was induced in the CS + LPS group, showed that GO and DADS treatment considerably inhibited the elevation of inflammatory cells and the production of IL-1β, IL-6, and TNF-α in the BALF, as well as the accumulation of inflammatory cells in the lung tissues. Furthermore, the treatment of GO and DADS markedly inhibited ERK phosphorylation and MMP-9 expression in the lung tissues, which were induced in the CS + LPS group.

CS acts as a potent stimulus that induces the inflammatory response because CS contains a large quantity of toxic chemicals. The exposure of the respiratory tract, in particular, to CS leads to the continuous infiltration of inflammatory cells into the CS-exposed lesions [2]. Of the infiltrated inflammatory cells, neutrophils act as important inducers for the pathological alteration of the respiratory tract. According to previous studies, the neutrophils that are activated by CS exposure release various stimulatory factors (including cytokines, chemokines, growth factors, chemoattractants, ROS, and proteases) that eventually increase inflammatory cell infiltration and aggravate the destruction of the normal alveolar structure [25,26]. Therefore, the reduction in the accumulation of neutrophils has been regarded as an important aspect of therapeutic strategies for treating pulmonary diseases that are induced by CS exposure. In this study, GO and DADS effectively suppressed the elevation of inflammatory cells induced in the BALF in the CS + LPS group, particularly of neutrophils. Consistent with these results, GO and DADS also inhibited the accumulation of inflammatory cells that were elevated in the lung tissues of the mice that were treated with CS + LPS. Based on these pieces of evidence, we conclude that GO and DADS could be potential preventive agents against CS-induced airway inflammatory diseases via the suppression of neutrophil infiltration.

Mitogen-activated protein kinases are regarded as critical limiting factors in the development of inflammatory responses. ERK, in particular, is phosphorylated in response to various stimuli that are involved in the transcription of inflammatory mediators, such as cytokines, chemokines, nitric oxide, and proteolytic enzymes [27]. These events finally lead to the development and aggravation of inflammatory responses. According to previous studies, CS acts as a potent inducer for ERK phosphorylation, leading to the production of proinflammatory cytokines as well as MMPs [28]. In various studies, the activation of ERK signaling has been shown to induce MMP-9 expression, which is abolished in the presence of an ERK inhibitor [29,30,31]. In particular, Su et al. [32] reported that DADS caused the suppression of the ERK/Fra-1 pathway, resulting in the downregulation of MMP-9 expression. MMP-9 is a proteolytic enzyme that is involved in the destruction of the normal alveolar structure, which results in the loss of normal lung function [2]. In this study, the exposure to CS and LPS caused elevation in the production of proinflammatory cytokines and MMP-9 with an increase in ERK phosphorylation. However, GO and DADS effectively suppressed these pathophysiological changes shown in the CS + LPS group. These results suggest that the preventive effects of GO and DADS on airway inflammation that has been induced by CS and LPS is closely related to the downregulation of ERK phosphorylation. These effects of GO and DADS are strongly supported by previous studies. The suppression of ERK phosphorylation by DADS is related to the protective effects against cyclophosphamide-induced hemorrhagic cystitis in rats and the inhibitory effects of TNF-α-induced production of monocyte chemotactic protein-1 in MDA-MB-231 cells [33,34]. In addition, Kuo et al. [35] reported that GO effectively protects against endotoxin-induced inflammatory responses via the suppression of neutrophil infiltration.

## 5. Conclusions

Overall, GO and DADS effectively inhibited the inflammatory cell infiltration and cytokine production caused by exposure to CS and LPS. In addition, GO and DADS downregulated ERK phosphorylation and MMP-9 expression. Therefore, our study suggests that GO and DADS could be potential preventive agents against inflammatory diseases that are induced by CS exposure.
